# Potential of Mobile LiDAR Sensors in Hiking Trail Management

**DOI:** 10.3390/s26165143

**Published:** 2026-08-14

**Authors:** Rui Fernandes, Alberto Gomes, Borja Moya-Gomez, Nelson Mileu

**Affiliations:** 1 Centre of Studies in Geography and Spatial Planning, University of Porto, 4150-564 Porto, Portugal; up200902906@up.pt; 2 Transport, Infrastructure and Territory Research Group (tGIS), Department of Geography, Complutense University of Madrid, 28040 Madrid, Spain; bmoya01@ucm.es; 3 Institute of Geography and Spatial Planning, University of Lisbon, 1600-276 Lisbon, Portugal; nmileu@edu.ulisboa.pt

**Keywords:** mobile LiDAR, hiking trails, trail monitoring, smartphones, trail degradation

## Abstract

**Highlights:**

**What are the main findings?**

- The evaluated smartphone LiDAR reproduced fine-scale hiking trail morphology of a short segment, including incision degree and pattern, exposed roots, and surface irregularities under dense canopy conditions.
- Continuous three-dimensional surface models supported exploratory identification of potential runoff pathways and relative surface depressions not fully captured by conventional discrete transect measurements.

**What are the implications of the main findings?**

- Smartphone-based LiDAR represents a rapid, accessible, and relatively low-cost alternative for hiking trail assessment and morphometric characterization.
- The results support further evaluation of smartphone LiDAR as a tool for localized trail monitoring and morphometric characterization in recreational environments.

**Abstract:**

The LiDAR sensor embedded in recent iPhone Pro devices offers new opportunities for rapid, low-cost, and spatially detailed assessment of outdoor recreational infrastructure. Framed within mobile sensing and IoT-based environmental monitoring, this proof-of-concept case study evaluates a smartphone-based LiDAR workflow for localized hiking trail assessment under dense canopy conditions. Four independent surveys of a degraded hiking trail section were conducted to assess inter-survey repeatability, agreement with conventional field measurements, and practical field applicability. The resulting three-dimensional models reproduced trail morphology, including incised tread sections, exposed roots, rocky surfaces, and localized irregularities relevant to trail-condition assessment. Repeated registrations demonstrated consistent cloud-to-cloud comparison metrics, while comparisons with field reference measurements showed good agreement in the representation of cross-sectional morphology and exposed root characteristics. Continuous surface reconstruction additionally supported exploratory identification of potential runoff pathways and relative surface depressions. Although limited to a single trail section and one smartphone–application combination (iPhone 13 Pro with Scaniverse), the evaluated workflow demonstrates potential as a rapid, accessible, and relatively low-cost approach for localized trail-condition assessment and provides a foundation for further evaluation in hiking trail monitoring.

## 1. Introduction

LiDAR sensors integrated into recent consumer-grade mobile devices have emerged as a low-cost and accessible alternative for high-resolution three-dimensional surveying [1,2,3]. Previous studies in geosciences, forestry, and object measurement have demonstrated that mobile device LiDAR can generate detailed and reliable surface models under suitable acquisition conditions [4,5,6,7,8]. Compared to conventional airborne or terrestrial laser-scanning systems, mobile device platforms substantially reduce equipment costs, operational complexity, and technical barriers, enabling rapid three-dimensional data acquisition by non-specialized users [3]. These characteristics create opportunities for exploring the application of mobile device LiDAR to localized environmental monitoring and surface characterization tasks that traditionally relied on more resource-intensive surveying approaches.

One potential application of this technology is the monitoring of hiking trail conditions. Hiking represents one of the most widespread forms of nature-based recreation, with increasing participation reported across protected areas and recreational landscapes worldwide [9,10,11]. Trails constitute the fundamental infrastructure supporting this activity, enabling visitor access while concentrating movement within designated corridors [12]. However, continued recreational use, combined with environmental factors such as precipitation, slope, soil type, root and rock protrusion, and runoff and drainage conditions, contributes to progressive trail degradation [11,13,14]. The monitoring of trail conditions therefore represents an essential component of sustainable trail management. Traditional assessment methods commonly rely on cross-sectional area measurements, in which a transect line is positioned across the trail and vertical measurements are manually collected relative to the tread surface [14,15]. These measurements enable the estimation of incision depth, widening, and soil loss [11,13,16,17]. Despite their widespread use and methodological simplicity, such approaches remain labor-intensive, spatially discontinuous, and dependent on the selection of representative transects [14]. Repeatability between surveys may additionally be influenced by differences in transect placement and operator decisions [15]. Consequently, conventional field surveys may provide spatially discontinuous representations of trail condition, limiting the characterization of fine-scale tread variability, localized surface transitions, and the continuous organization of trail morphology along degraded segments. This is particularly apparent across extensive trail networks where systematic monitoring becomes difficult due to time, staffing, and financial constraints.

Recent advances in remote sensing have enabled the use of high-resolution topographic data for trail assessment and erosion monitoring. Airborne LiDAR and Unmanned Aerial Vehicle (UAV) based surveys have demonstrated their usefulness for identifying trail incision, erosion patterns, and surface deformation [18,19,20,21,22,23]. These approaches provide detailed terrain representations and support spatially continuous analyses that are difficult to achieve through traditional transect-based methods. However, their application often requires specialized equipment, trained personnel, flight operations, and relatively high operational costs [18,23]. In densely vegetated environments, photogrammetric reconstruction may additionally be constrained by canopy cover and limited visibility of the trail surface [18]. As a result, the implementation of these methods for frequent monitoring of recreational trail networks remains challenging for many management authorities. Despite these advances, the application of mobile device LiDAR to direct trail-condition assessment remains comparatively unexplored, particularly for fine-scale tread characterization under densely vegetated conditions.

This study presents a proof-of-concept evaluation of a consumer smartphone LiDAR sensor for the three-dimensional characterization of hiking trail conditions under dense forest canopy. Specifically, the study pursues three main objectives: (i) to assess the repeatability of repeated smartphone LiDAR surveys and their agreement with conventional field measurements; (ii) to explore the capability of mobile device LiDAR to characterize trail incision, exposed roots, and localized surface irregularities relevant to trail-condition assessment; and (iii) to assess the practical feasibility of using this workflow for rapid field documentation of hiking trail conditions under densely vegetated conditions. Rather than providing a general validation of consumer mobile LiDAR, this case study examines whether an iPhone 13 Pro using the Scaniverse application can produce repeatable, high-resolution representations of a short trail section that are sufficiently consistent with conventional field measurements to justify further investigation of the approach for trail-condition assessment.

## 2. Materials and Methods

### 2.1. Study Area

**Figure 1:**
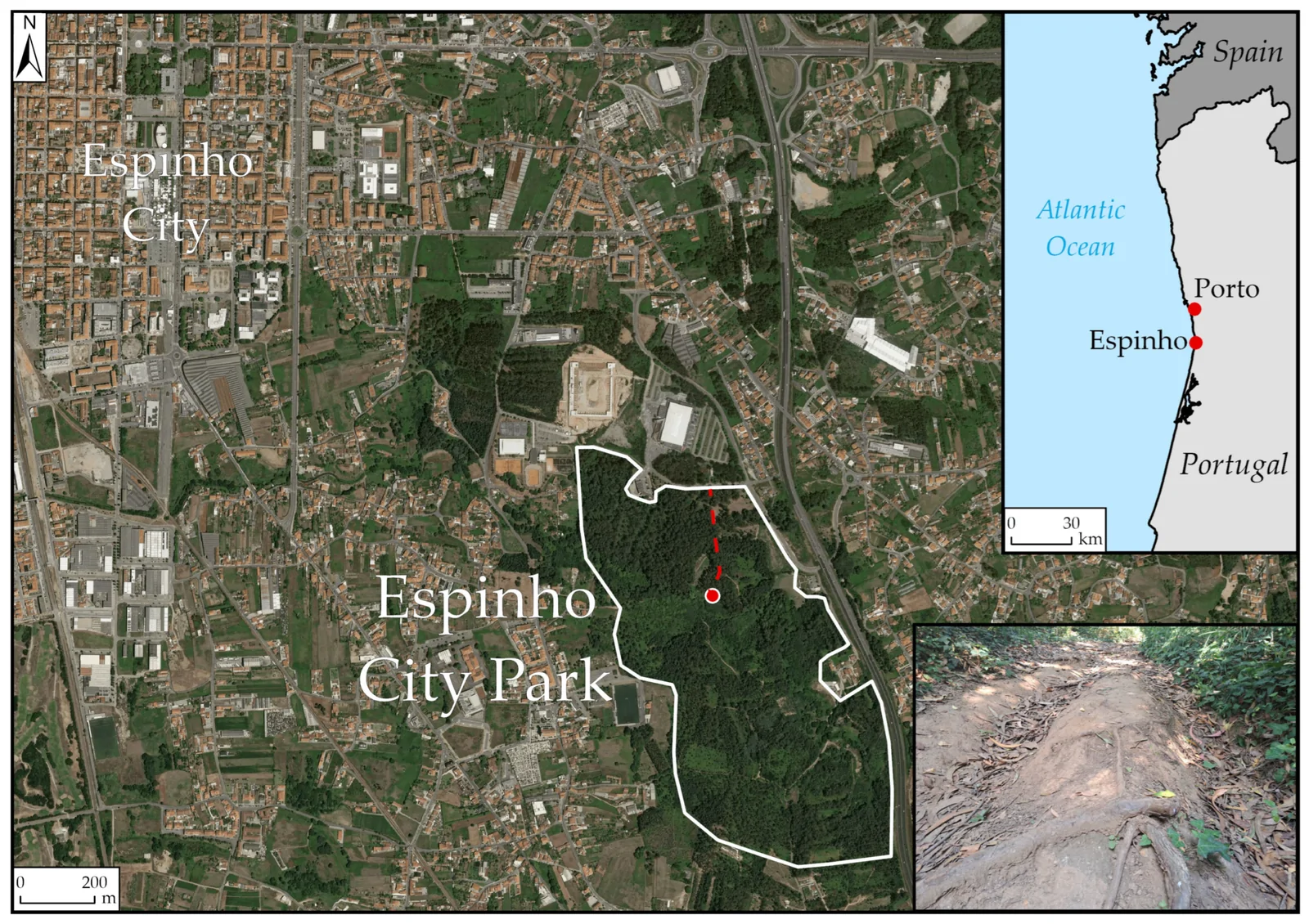
Study area. Location of the city park (southeast) and its trail network. The red line highlights the studied trail, and the red dot indicates the specific survey location.

This case study was conducted in Espinho City Park, a small town located in the municipality of Espinho, northwestern Portugal, approximately 20 km south of Porto (Figure 1). The park is included in the main green area of the municipality and contains an official network of three hiking trails [24]. These trails are accompanied by numerous informal and recreational trails used for pedestrian circulation and outdoor leisure activities. The selected trail segment is located within a densely vegetated area characterized by partial to closed canopy cover. The segment follows a roughly North–South orientation, with a mean slope of 17.3°.

The selected trail section exhibited visible signs of degradation associated with concentrated pedestrian and cycling use, including tread incision, exposed roots, uneven surface morphology, and localized erosion features (Figure 1). The presence of pronounced micro-topographic irregularities and exposed roots made the site suitable for evaluating the capability of the tested smartphone LiDAR workflow to capture fine-scale trail-surface characteristics.

The dense arboreal cover present along the surveyed trail segment additionally limited the acquisition of reliable satellite-based positioning data. Attempts were made to record ground-control coordinates using an Emlid Reach RX (Emlid Tech Kft., Budapest, Hungary) high-precision GNSS receiver with differential correction; however, canopy obstruction prevented stable satellite reception and consistent positional correction within the study area. As a result, the individual LiDAR surveys exhibited small positional and rotational discrepancies prior to alignment, requiring subsequent manual registration during preprocessing. Consequently, the repeated surveys could not be directly compared within a common geodetic reference frame, and the evaluation focused on the repeatability of the registered point clouds and their agreement with conventional field measurements rather than on absolute geospatial accuracy. Field surveys and manual measurements were conducted along a representative trail segment approximately 2 m wide and between 8 and 10 m long, depending on the survey. The limited spatial extent was intentionally selected to provide a controlled test area containing a range of trail-surface conditions, including relatively smooth tread sections and highly irregular areas with exposed roots and localized erosion.

### 2.2. Mobile LiDAR Acquisition

Four independent mobile LiDAR surveys were conducted using the integrated LiDAR sensor of an iPhone 13 Pro. The device employs a near-infrared Vertical Cavity Surface Emitting Laser (VCSEL) technology [7], enabling the acquisition of dense three-dimensional point clouds at short ranges. Data collection was performed using the Scaniverse iOS v5.2.5 application (https://dev.scaniverse.com), which interfaces directly with the device’s LiDAR camera to generate textured three-dimensional reconstructions and point clouds. This sensor has a reported maximum range of 5 m [7,8], which makes it suitable for these types of survey.

Surveys were performed by two operators under similar environmental conditions but with variations in acquisition direction, segment length, phone orientation, and approximate sensor-to-surface distance. Surveys were performed by manually walking along the trail while continuously scanning the tread surface and adjacent terrain. Data acquisition focused on maximizing surface coverage and maintaining visibility of localized trail features, including incised sections and exposed roots. Slight variations between surveys were intentionally permitted to represent realistic field acquisition conditions rather than a strictly controlled laboratory protocol. The approximate sensor-to-surface distance reported in Table 1 was visually estimated during field acquisition based on the operator’s scanning posture and the position of the smartphone relative to the trail surface. Surveys performed while standing with the device held near chest or head height were classified as having an approximate sensor-to-surface distance greater than 1 m, whereas surveys conducted while crouching and holding the device closer to the trail surface were classified as having an approximate distance below 1 m. The reported values therefore represent broad acquisition categories rather than direct field measurements.

Initial inspection of the resulting point clouds revealed small positional offsets and rotational differences between surveys, particularly between horizontally and vertically acquired scans. These differences were likely associated with mobile device SLAM reconstruction and reduced GNSS reliability beneath dense canopy cover [21,25]. Point clouds were therefore imported into the CloudCompare v2.13.2 software (https://www.cloudcompare.org) for preprocessing. Alignment consisted of an initial coarse manual rotation and translation to achieve approximate overlap, followed by Iterative Closest Point (ICP) registration to optimize the rigid transformation. The aligned point clouds were subsequently cropped to their common overlapping extent prior to cloud-to-cloud comparison.

To evaluate the influence of the manual registration procedure, the complete alignment workflow (manual coarse alignment followed by ICP refinement) was independently repeated three times for each survey pair. Cloud-to-cloud distances were calculated after cropping the point clouds to their common extent, and points exceeding the 95th percentile of distances were excluded to reduce the influence of isolated outliers and edge artifacts. Summary statistics from the repeated registrations were used to quantify the repeatability of the registration workflow.

**Figure 2:**
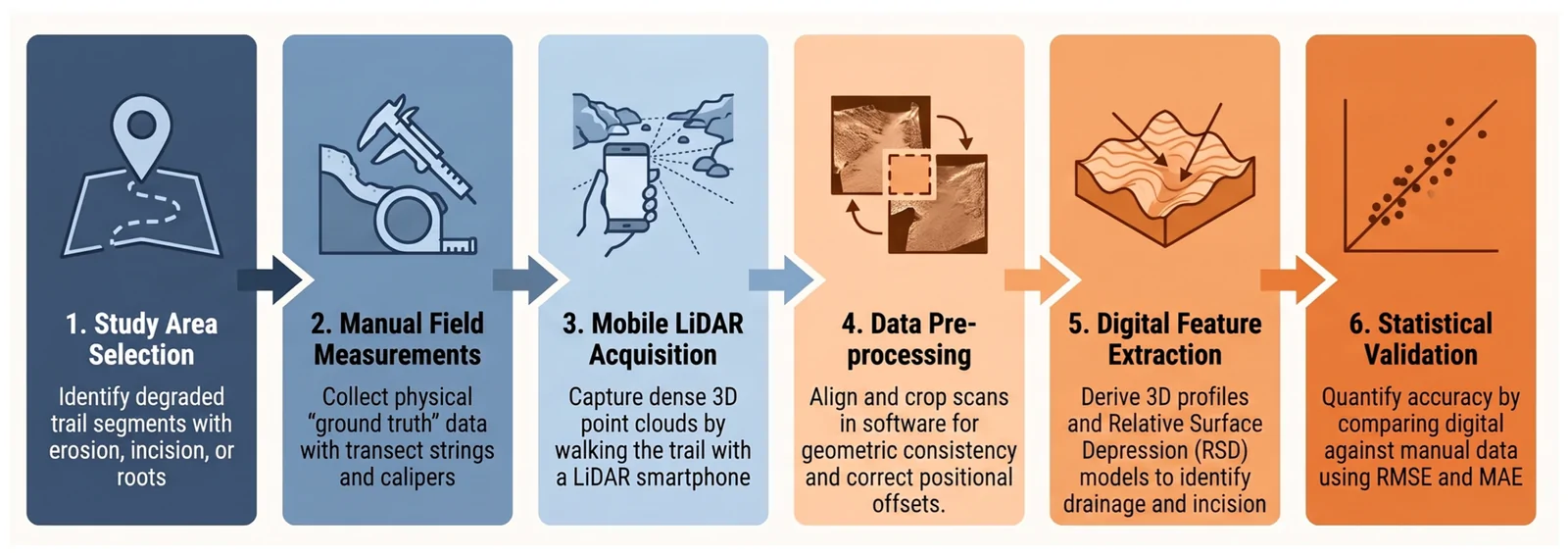
Workflow of the study, from location selection to data validation.

Figure 2 shows the workflow of the study.

### 2.3. Manual Field Measurements

**Figure 3:**
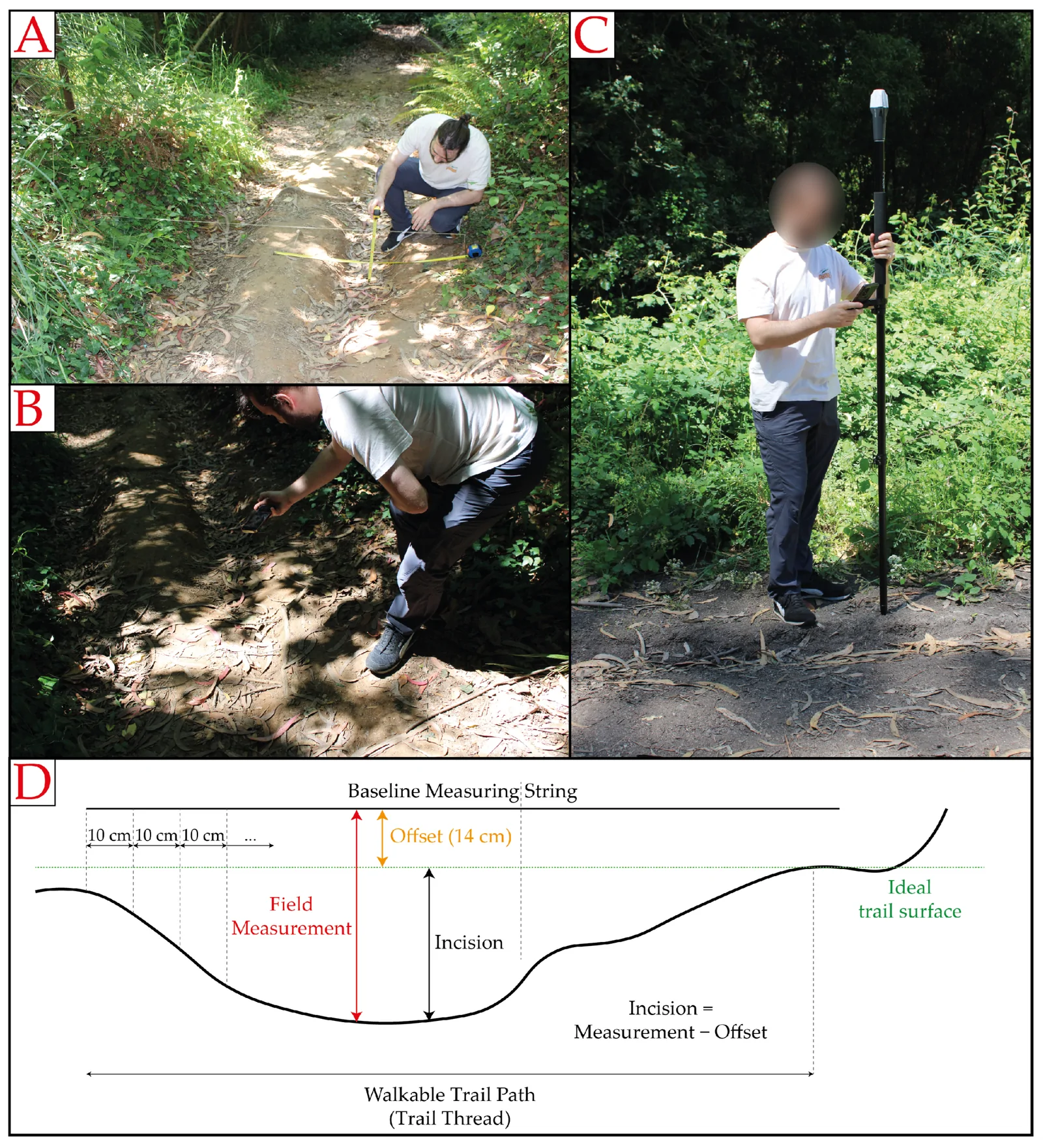
Measuring methods in the field. (**A**) A string attached to two supports serves as a baseline for the cross-section method. (**B**) LiDAR survey using a mobile device. (**C**) Unsuccessful attempts to acquire precise GPS coordinates. (**D**) Conceptualization of the field measurements visible in (**A**). Measurements were done every 10 cm along the transect.

Manual field measurements were conducted as the reference method for evaluating LiDAR-derived trail morphology and localized surface features. Measurements focused on two main components: trail cross-sectional morphology and exposed root characterization (Figure 3). An attempt to use a high-precision GPS was made, with negative results.

Trail cross-sectional measurements were obtained using a tensioned reference string positioned horizontally across the trail tread and fixed to two lateral support points located outside the main incised surface. Vertical distances between the string and the trail surface were measured at regular 10 cm intervals using a tape measure. The resulting profile therefore represents a discrete sampling of the trail cross-section, with the intervening surface inferred between measurement locations. These measurements represented relative incision depth and cross-sectional morphology along the surveyed trail profile.

Exposed root measurements were performed along a representative exposed root feature intersecting the trail tread. Eight transverse transects were established perpendicular to the root axis. Root protrusion height relative to the adjacent tread surface was measured using a tape measure, while root width was measured using a caliper. Root length was additionally recorded along its visible extent. These measurements provided reference dimensions for evaluating the LiDAR-derived representation of localized surface irregularities relevant to trail accessibility and tread degradation.

### 2.4. Digital Measurements and Profile Extraction

LiDAR-derived measurements were obtained from the processed and registered point cloud models using GIS (ArcGIS Pro 3.6.2) and point cloud analysis software. Cross-sectional and root profiles were extracted directly from the digital surface representation at positions corresponding to field-measurement locations.

Cross-sectional profiles were converted to relative elevation values using the least-incised portion of the trail tread as the local reference surface. This normalization procedure allowed direct comparison between field-derived incision measurements and LiDAR-derived surface morphology.

Root profiles were extracted across the same eight transects used during field measurements. Width and protrusion measurements were obtained from digital profiles using analogous geometric criteria to those applied in the field. Additional qualitative observations regarding surface morphology, localized roughness, and vegetation interference were also recorded during profile interpretation. In addition to profile extraction, the LiDAR-derived surface models were also used for exploratory drainage analysis. An exploratory drainage analysis was conducted to examine whether the LiDAR-derived surface models could identify potential runoff pathways and areas susceptible to water concentration along the trail tread. A Relative Surface Depression (RSD) model was additionally derived from the digital surface model to identify localized topographic depressions and microtopographic confinement zones along the trail tread. The RSD representation was used to support the interpretation of potential runoff pathways and the spatial relationship between surface morphology and drainage organization.

### 2.5. Statistical Analysis

Cloud-to-cloud comparisons were used to evaluate the repeatability of repeated LiDAR surveys, whereas comparisons with manual field measurements were used to assess agreement between the LiDAR-derived and conventional measurement methods.

Inter-survey repeatability was evaluated through cloud-to-cloud distance analyses for all survey pairs following three independent repetitions of the alignment workflow. For each survey pair, the mean cloud-to-cloud distance, Root Mean Square Error (RMSE) [26,27], 95th percentile distance, and the proportion of points with cloud-to-cloud distances below 2 cm were calculated. The reported values correspond to the mean and standard deviation of the three independent registrations, while the relative range between repeated registrations was additionally calculated to quantify the sensitivity of each metric to repeated alignment.

Agreement between LiDAR-derived measurements and field reference measurements was performed using Mean Absolute Error (MAE), RMSE, Mean Bias, Pearson correlation coefficient, and maximum absolute error, following Chai and Draxler [26] and Willmott and Matsuura [27]. These metrics were used to quantify the magnitude and direction of the differences between the two measurement approaches.

Profile morphology and localized surface irregularities were additionally assessed qualitatively through visual comparison between field observations and LiDAR-derived profiles.

## 3. Results

Four independent mobile LiDAR surveys produced dense three-dimensional representations of the selected trail segment despite differences in acquisition direction, phone orientation, and survey extent. The following sections present the preprocessing and alignment of the surveys, the repeatability of repeated cloud-to-cloud comparisons, the agreement between LiDAR-derived and field reference measurements, and the exploratory terrain analyses derived from the resulting surface models. Together, these analyses evaluate the repeatability of the smartphone LiDAR workflow, its agreement with conventional field measurements, and its capability to represent trail-surface characteristics within the scope of this case study.

### 3.1. Survey Characteristics and Spatial Consistency

**Figure 4:**
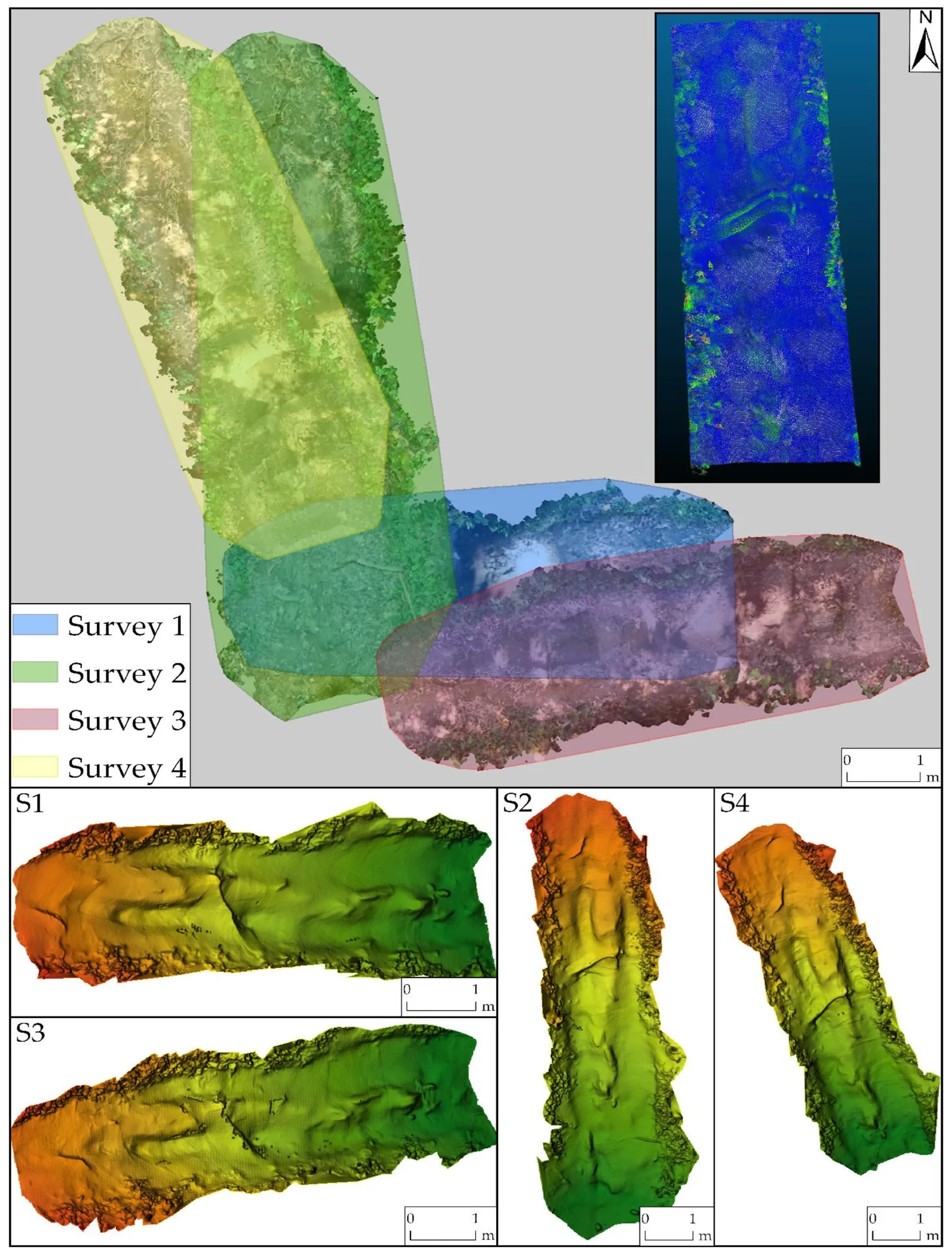
Spatial overlap and orientation differences between the four mobile LiDAR surveys. The upper-left panel shows the overlap of the four surveys, with transparent polygons outlining their spatial extents. The upper-right inset illustrates the aligned point clouds in CloudCompare. The lower panels (S1–S4) present the digital surface models (DSMs) derived from each survey.

The four mobile LiDAR surveys exhibited noticeable differences in spatial orientation, extent, and raw positioning prior to alignment (Figure 4). Variations were particularly evident between surveys acquired using horizontal and vertical phone orientations. Vertical surveys preserved the original North–South orientation, while horizontal ones rotated the surveys 90°. This resulted in surveys 1 and 3 having East–West orientation, while being in the correct spot and maintaining correct measures (Figure 4).

Although all surveys contained embedded geographic positioning derived from the mobile device, the dense arboreal cover prevented the acquisition of reliable differential GNSS control points within the surveyed segment. Attempts to obtain high-precision ground control using an Emlid Reach RX receiver were unsuccessful due to unstable satellite reception beneath canopy cover. Consequently, the original positioning associated with the LiDAR surveys was insufficient to guarantee direct point-to-point correspondence between point clouds. The exported point clouds nevertheless preserved the original local orientation associated with each individual acquisition sequence. As a result, surveys acquired using different phone orientations and movement trajectories exhibited distinct relative coordinate frames and rotational offsets prior to alignment (Figure 4).

Differences in survey extent were additionally observed between operators, resulting in slight variations in total surveyed length and peripheral coverage. Nevertheless, the central portion of the analyzed trail segment was consistently captured across all surveys. Because of the combined effects of positional deviation, rotational inconsistencies, and differing survey extents, direct geometric comparison between raw point clouds proved difficult prior to preprocessing.

Manual alignment followed by ICP registration and cropping procedures were therefore required in order to establish geometric correspondence between surveys. Alignment focused on maximizing overlaps within the central trail corridor and minimizing rotational discrepancies between the principal tread features. Despite the observed inconsistencies, all surveys reproduced the overall morphology of the analyzed trail segment, including the main tread geometry, incised sections, exposed roots, and adjacent vegetation structure (Figure 4). Survey overlap remained substantial across the central portion of the trail, allowing subsequent cloud-to-cloud comparison and profile extraction following alignment procedures.

Table 1 summarizes the main acquisition characteristics of each survey. Surveys performed by User 2 required longer acquisition times, although this difference was partially associated with slightly larger surveyed extents beyond the main analysis segment. Consequently, acquisition duration could not be directly related to survey quality or geometric consistency. Similarly, no clear relationship was observed between survey direction and resulting cloud consistency.

Visual inspection of the aligned point clouds indicated that surveys acquired using horizontal phone orientation generally presented lower rotational discrepancies relative to the trail axis and required less manual adjustment during preprocessing. Conversely, vertically acquired surveys exhibited greater orientation differences and slightly more variable spatial positioning. However, all surveys remained sufficiently coherent following alignment to permit quantitative comparison and morphometric analysis.

The repeated registration analysis workflow implemented in CloudCompare successfully reduced positional discrepancies between surveys and standardized overlapping extents for subsequent analysis (Figure 4). This procedure allowed the comparison to focus primarily on relative geometric consistency and local trail morphology rather than on absolute geospatial positioning, which was affected by canopy-related GNSS limitations during acquisition.

### 3.2. Inter-Survey Reproducibility

Cloud-to-cloud comparison between the four mobile LiDAR surveys demonstrated good repeatability across three independent repetitions of the alignment workflow (Table 2), with the results of the individual repetitions provided in Tables S1–S3. Mean cloud-to-cloud distances ranged from 1.00 ± 0.17 cm to 1.90 ± 0.41 cm, while RMSE values ranged from 1.07 ± 0.22 cm to 2.39 ± 0.56 cm.

The smallest discrepancies were consistently observed for the S1–S2 comparison, which presented the lowest mean distance (1.10 ± 0.20 cm) and RMSE (1.07 ± 0.22 cm). Comparisons involving Survey 4 exhibited the largest discrepancies across all involved pairs, although the variability between repeated registrations remained modest relative to the absolute cloud-to-cloud distances. These differences were likely associated with variations in acquisition orientation, sensor-to-surface distance, and localized positioning inconsistencies during data capture.

Relative ranges varied between 12.7% and 38.9% for the mean cloud-to-cloud distance, and between 23.0% and 44.8% for RMSE. Although these percentages appear relatively large, they correspond to absolute differences of only a few millimetres between repeated registrations.

The 95th percentile distances ranged from 2.76 ± 0.22 cm to 8.10 ± 1.76 cm. Survey pairs involving Survey 4 consistently exhibited higher 95th percentile values, indicating a greater proportion of larger local discrepancies despite similar overall patterns of agreement. Between 63.9% and 89.4% of points remained within 2 cm across the repeated registrations. The highest proportions were observed for S1–S2 and S1–S3, whereas comparisons involving Survey 4 consistently showed lower proportions of points below this threshold. No clear relationship was identified between acquisition duration and resulting geometric consistency. Although surveys acquired by Author 2 required longer acquisition times, these surveys also covered slightly larger spatial extents beyond the primary analysis segment. Similarly, survey direction alone did not appear to substantially influence repeatability. However, surveys acquired using horizontal phone orientation generally exhibited lower discrepancies relative to vertically acquired surveys, suggesting that device orientation may influence the stability of mobile device SLAM reconstruction under field conditions.

The repeatability analysis indicates that the manual registration workflow produced consistent cloud-to-cloud comparison statistics across independent repetitions. Although survey pairs involving Survey 4 consistently exhibited larger discrepancies, the between-registration variability remained small in absolute terms, supporting the use of the proposed workflow for repeatable characterization of trail-surface morphology within the scope of this case study.

### 3.3. Cross-Sectional Analysis and Validation

**Figure 5:**
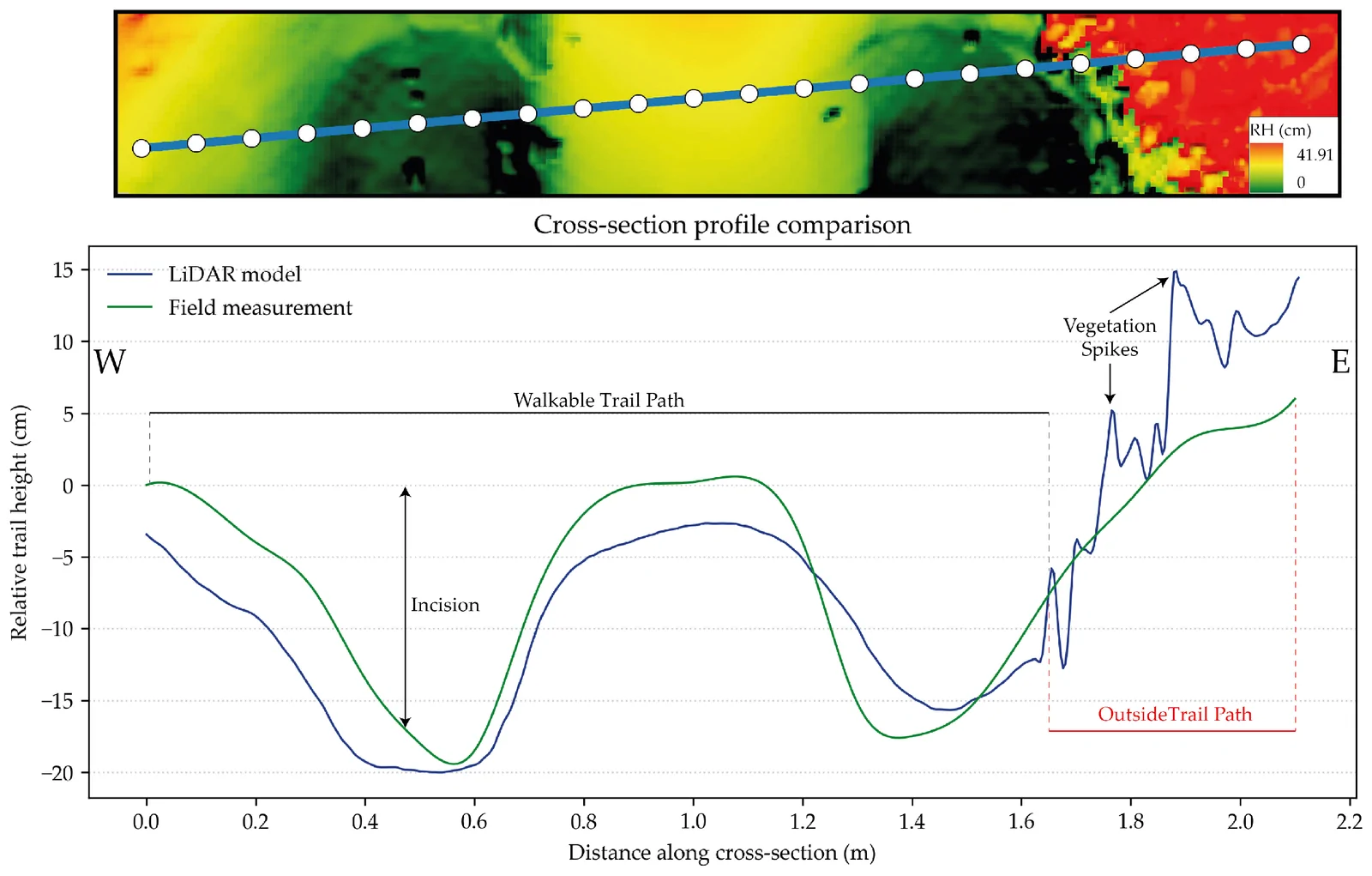
Comparison between LiDAR-derived and field reference trail cross-sectional profiles, highlighting incision morphology, walkable tread limits, and vegetation-related irregularities. Above is the DSM with the points where the field measurements were taken, approximately aligned with the graph cross-section.

LiDAR-derived cross-sectional measurements showed good overall agreement with the manually measured trail profile. The reconstructed profile reproduced the principal morphological characteristics of the surveyed trail section, including the two main incised zones, the elevated central portion associated with exposed rock, and the overall asymmetry of the tread surface (Figure 5). The general W-shaped morphology observed during field measurements was consistently represented in the LiDAR-derived model.

The surveyed trail section exhibited a distinct W-shaped morphology characterized by two primary incised zones located laterally to a slightly elevated central portion associated with exposed rock. Rather than presenting a single central incision channel, the trail tread was divided into two preferential walking corridors positioned on either side of the exposed rocky surface. This configuration likely reflects repeated visitor avoidance of the central obstacle, resulting in localized concentration of foot traffic and differential tread erosion along the lateral portions of the trail.

Both field and LiDAR-derived profiles consistently reproduced this general morphology. The LiDAR-derived model accurately identified the position and relative depth of the two main incised sections, as well as the abrupt transitions associated with exposed rock and tread-edge boundaries. Minor differences between methods were primarily associated with localized smoothing of sharp surface transitions and increased irregularity near vegetated trail edges. Nevertheless, the overall spatial distribution of incision and tread morphology remained coherent between both datasets.

Comparison between LiDAR-derived and field reference values yielded an MAE of 4.11 cm and an RMSE of 4.78 cm for the complete transect (Table 3). Pearson correlation between both datasets reached 0.86, indicating strong agreement in overall geometry and relative surface variation. The maximum absolute discrepancy observed along the transect was 9.69 cm and occurred near the transition between the walkable tread and the adjacent vegetated edge.

The LiDAR-derived profile consistently represented greater incision depths than those obtained from field reference measurements, resulting in a positive Mean Bias of 4.11 cm. In the profile visualization (Figure 5), this behavior appears as a systematic lowering of the LiDAR-derived surface relative to the manually measured profile. This offset was particularly evident along the main incised sections of the trail. Discrepancies were greater outside the effective walkable trail tread than within it (Table 3), suggesting that vegetation and irregular trail margins contributed to the observed differences. However, a positive bias of 3.83 cm remained within the walkable trail surface, indicating that vegetation alone does not fully explain the systematic offset. Additional contributions may arise from differences in spatial sampling, as the field profile was measured at discrete 10 cm intervals whereas the LiDAR-derived profile provides a continuous representation of the trail surface. The present dataset does not allow the individual contributions of these factors to be isolated.

The section corresponding to the effective walkable trail tread exhibited slightly lower discrepancies than the complete transect, with MAE and RMSE values decreasing to 3.83 cm and 4.58 cm, respectively. This portion of the profile was comparatively less affected by vegetation interference and contained smoother transitions between tread surfaces. Pearson correlation remained high (0.81), indicating that the principal morphology and relative depth distribution of the trail incision were consistently represented within the actively used trail corridor.

The outer portion of the profile, located beyond approximately 1.6 m along the transect, corresponded to terrain outside the effective walkable trail tread. In this section, the LiDAR-derived profile exhibited abrupt spikes and localized irregularities associated with overhanging vegetation captured during acquisition (Figure 5). This area presented slightly higher MAE and RMSE values, reaching 5.08 cm and 5.39 cm, respectively. Nevertheless, Pearson correlation increased to 0.95, indicating that despite larger localized deviations, the broader terrain morphology and relative elevation variability remained strongly coherent between both datasets.

Visual inspection of the continuous LiDAR-derived profile additionally revealed localized elevation spikes exceeding the maximum discrepancies represented in the statistical comparison, particularly near vegetated trail margins. These features were not fully reflected in the quantitative metrics because field measurements were acquired at discrete 10 cm intervals rather than continuously along the transect. As a result, some abrupt microtopographic irregularities captured by the LiDAR reconstruction were located between sampled field points and therefore were not directly incorporated into the point-by-point statistical comparison. This highlights an important distinction between conventional transect measurements and continuous three-dimensional surface reconstruction, as the LiDAR-derived model preserved fine-scale terrain variability and localized surface transitions that remained undetected between discrete field sampling intervals.

Despite localized discrepancies, the LiDAR-derived model preserved the general geometry and relative depth distribution of the trail incision. The reconstructed profile clearly differentiated between the walkable tread surface and adjacent non-tread terrain. These results indicate that the tested workflow can reproduce the principal cross-sectional morphology of the surveyed trail section despite localized discrepancies associated with vegetation and profile sampling.

### 3.4. Root and Localized Surface-Feature Characterization

**Figure 6:**
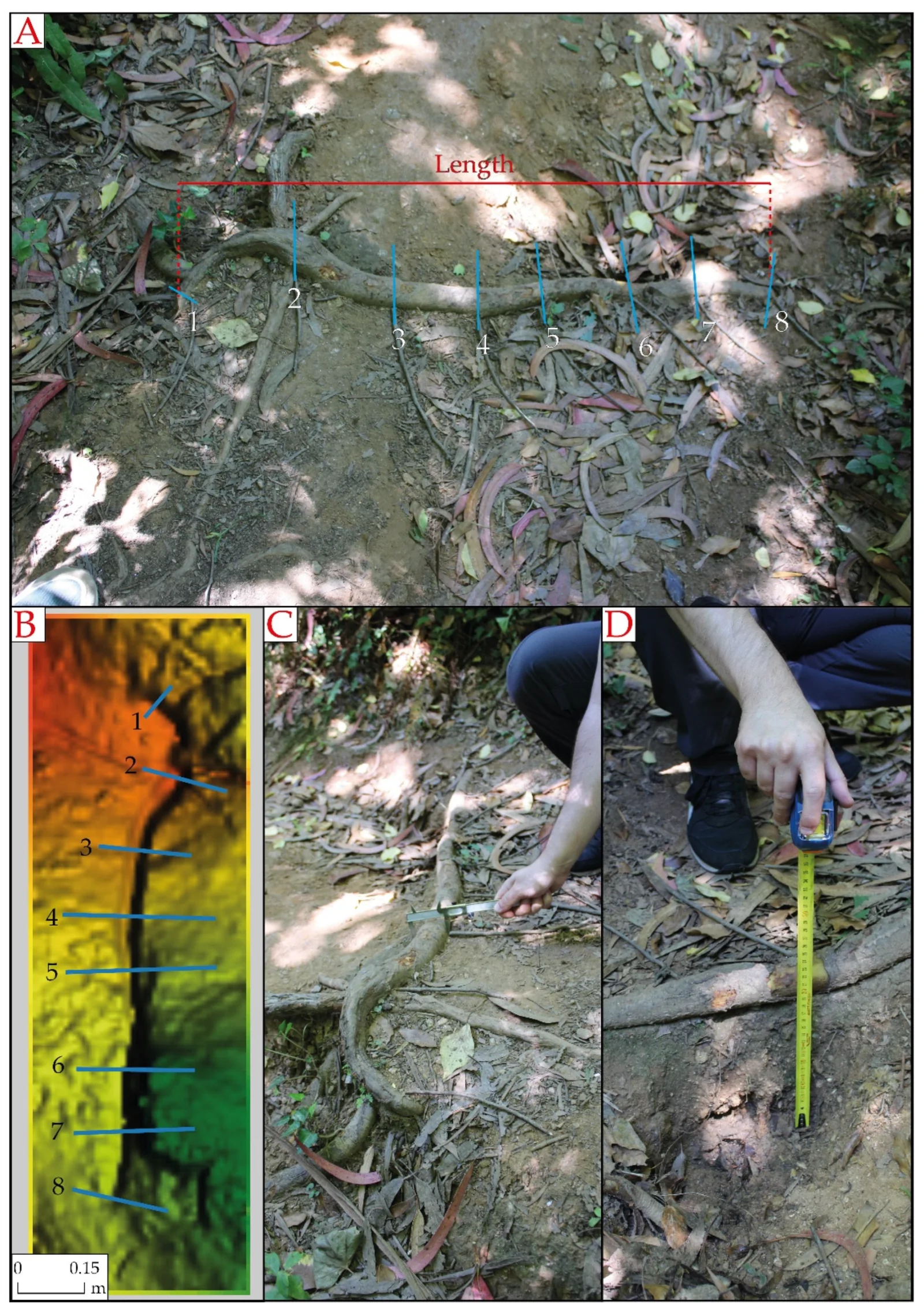
Root features analyzed in the study and associated field measurement procedures. (**A**) Exposed root section showing the measured root length and the eight transects used for root width and protrusion. (**B**) DSM of the root feature with the eight transects used for profile extraction and comparison. (**C**) Field measurement of root width using a caliper. (**D**) Field measurement of root protrusion relative to the trail surface using a tape measure.

Localized root morphology extracted from the LiDAR-derived model showed good agreement with field measurements across the eight analyzed transects (Figure 6). The reconstructed profiles reproduced the general geometry of the protruding root, including abrupt surface transitions, asymmetric protrusion geometry, and variations in root exposure along its length. The profiles also captured changes in protrusion intensity between transects, with the root becoming progressively more exposed toward the central section of the feature.

Width measurements derived from the LiDAR model showed comparatively low discrepancies relative to field observations (Table 4). MAE and RMSE values for root width were 0.53 cm and 0.56 cm, respectively, indicating good agreement between the LiDAR-derived and field reference measurements. Relative width errors remained low for most transects, generally below 15%, and the LiDAR-derived profiles consistently reproduced the lateral extent of the exposed root surface. The smallest discrepancies were observed in transects characterized by smoother transitions between the root and adjacent tread surface.

Protrusion measurements exhibited greater variability than width measurements. MAE and RMSE values for protrusion height reached 2.88 cm and 3.95 cm, respectively, indicating that a limited number of larger discrepancies contributed disproportionately to the overall error. The largest discrepancies occurred in transects associated with steeper surface transitions and greater protrusion heights, particularly in the central portion of the root where incision adjacent to the root was more pronounced. In several cases, the LiDAR-derived model represented greater protrusion heights than those measured in the field. This pattern may reflect localized amplification of sharp microtopographic transitions during surface reconstruction, although the available data does not allow this effect to be distinguished from other sources of measurement disagreement.

**Figure 7:**
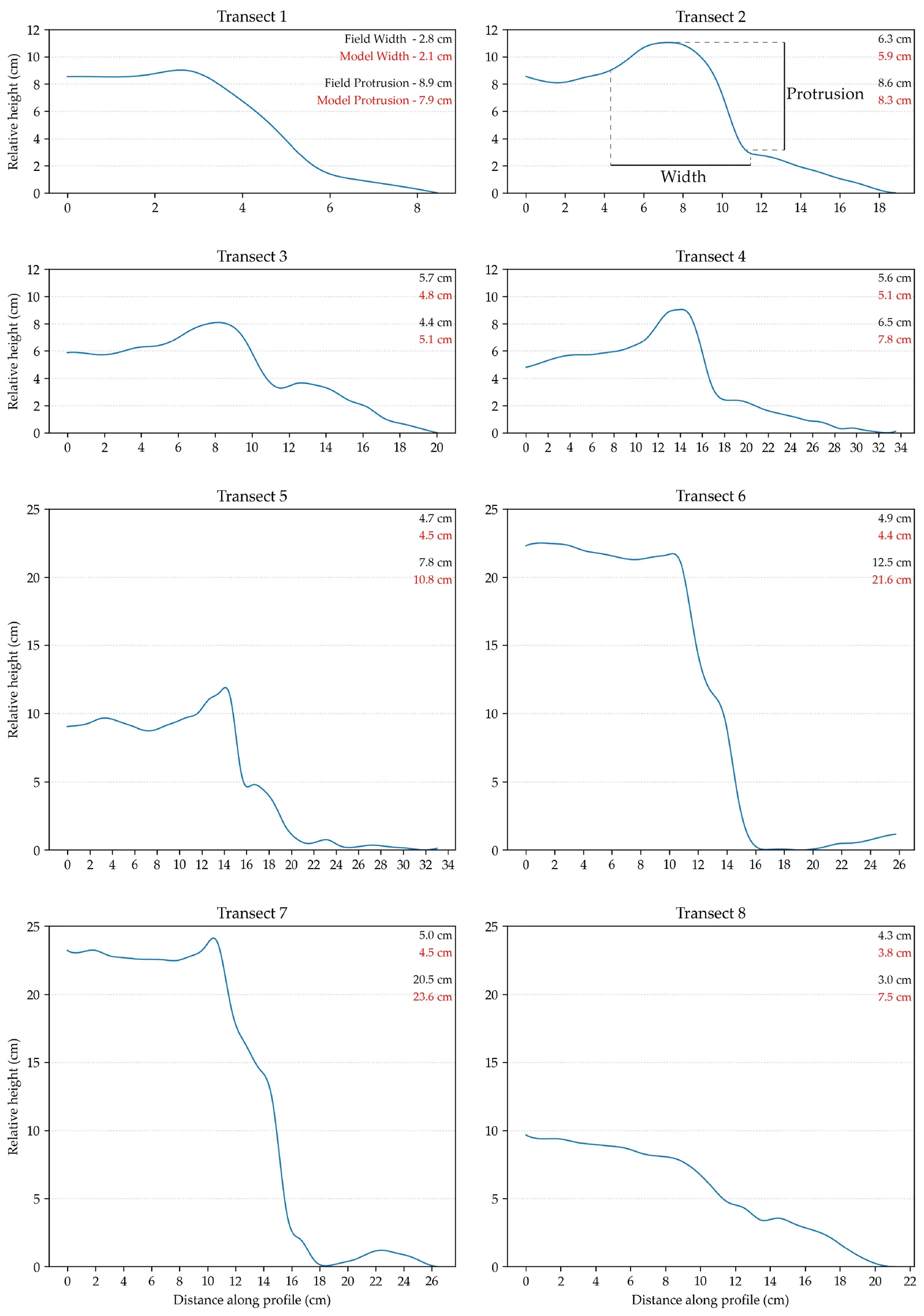
LiDAR-derived transverse profiles of the exposed root system across eight transects, including field and model measurements of root width and protrusion.

Despite these localized differences, the LiDAR-derived profiles consistently preserved the overall morphology and spatial variability of the exposed root. The reconstructed transects clearly differentiated between low-relief and highly protruding sections of the root and reproduced the progressive increase in protrusion toward the central portion of the feature (Figure 7). These results indicate that the tested smartphone LiDAR workflow was capable of capturing fine-scale trail-surface irregularities within the surveyed trail section, including localized obstacles associated with exposed roots and uneven tread morphology.

### 3.5. Drainage Pattern

**Figure 8:**
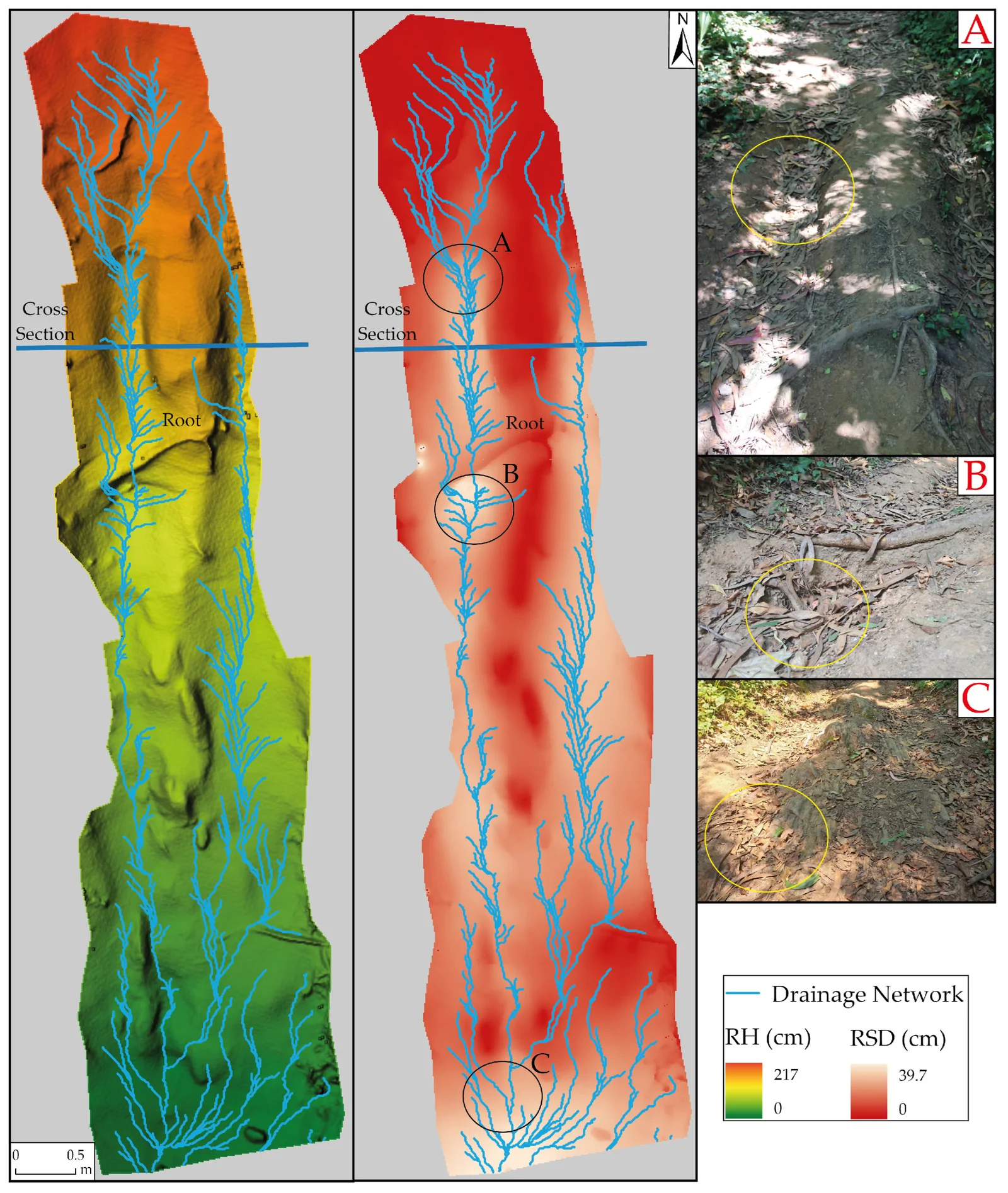
Drainage network and RSD derived from the DSM of Survey 2. The model was clipped to the trail path to avoid side vegetation interference. (**A**,**B**) show areas of increased incision after a sudden drop. (**C**) shows the increased incision on the left side of the path, diverting the drainage to the area. Yellow circles in the photos correspond to the matching black circles labelled with the same letters.

The digital surface model derived from the LiDAR survey enabled the extraction and visualization of localized potential runoff pathways along the analyzed trail segment (Figure 8). The derived potential runoff pathways predominantly followed the lower portions of the trail tread, forming continuous potential flow paths associated with the principal incised sectors of the trail. The drainage lines were concentrated along two preferential pathways located on either side of a more elevated and resistant central surface, producing a distinct dual-channel pattern across much of the surveyed segment. Additional secondary drainage branches were also identified near the trail margins and adjacent microtopographic depressions.

The RSD model revealed a spatial pattern largely consistent with the extracted drainage network. Higher RSD values were concentrated within elongated depressions corresponding to the most incised portions of the trail tread, while lower values occurred over the central elevated surface and less degraded sectors. The central portion of the trail was characterized by a relatively stable rocky surface that locally interrupted and diverted the potential flow paths. Further downslope, the RSD model indicated an increase in depression intensity toward the western side of the trail, where the drainage network progressively converged and shifted laterally in the same direction. Note the very depressed terrain just south of the root, to the left, corresponding to the sudden drops seen on transects 6 and 7 of Figure 6.

The relationship between the derived potential runoff network and the RSD model suggests a correspondence between surface morphology and preferential runoff concentration zones. Areas characterized by deeper relative depressions generally coincided with more continuous and organized potential flow paths, whereas sectors with reduced topographic confinement displayed more fragmented or diffuse drainage patterns. Localized deviations in drainage direction were additionally observed near exposed roots, abrupt slope transitions, and irregular surface features, suggesting that small-scale terrain variability may influence the organization of potential surface runoff across the trail tread.

## 4. Discussion

### 4.1. Mobile LiDAR Sensing for Hiking Trail Assessment

The results indicate that the tested smartphone LiDAR workflow was capable of producing morphologically coherent representations of hiking trail surface conditions under densely vegetated field environments. Despite variations in acquisition direction, phone orientation, and sensor-to-surface distance, the surveys maintained substantial overlap and relatively low inter-survey discrepancies following alignment and pre-processing. These results suggest that the tested device–application combination can generate sufficiently stable three-dimensional representations for localized trail-surface assessment under the conditions examined in this case study.

The findings are consistent with previous studies demonstrating the applicability of LiDAR-based approaches for trail and erosion monitoring [16]. Airborne and UAV-based surveys have previously been shown to effectively identify trail incision, erosion patterns, and surface deformation [16,18,19,20,21,23]. However, the present study complements these approaches by demonstrating that the evaluated smartphone LiDAR workflow can also reproduce relevant trail-surface morphology at fine spatial scales under realistic field conditions. While airborne and UAV-based methods provide broader spatial coverage and potentially greater positional accuracy, they frequently require specialized equipment, trained personnel, flight operations, and relatively high operational costs [18,23]. In contrast, mobile device LiDAR acquisition was rapid, required minimal equipment, and could be performed directly from the trail surface without specialized surveying procedures, as also observed in other mobile device LiDAR applications [3,5].

These characteristics may be particularly advantageous beneath partial or closed canopy, where photogrammetric reconstruction and GNSS-based positioning frequently become more challenging [18]. In the present study, dense vegetation cover prevented the acquisition of reliable differential GNSS correction despite attempts using a survey-grade receiver, requiring subsequent manual alignment of the point clouds. Nevertheless, repeated registrations produced consistent cloud-to-cloud comparison statistics, indicating that the manual registration workflow introduced only limited variability relative to the observed inter-survey differences. The repeatability analysis further demonstrated that repeating the complete manual alignment and ICP registration workflow produced only modest variation in the cloud-to-cloud comparison statistics, supporting the robustness of the adopted preprocessing procedure despite the absence of survey-grade georeferencing. At the same time, the study also highlighted important limitations associated with mobile device acquisition. Surveys exhibited small rotational and positional inconsistencies prior to alignment, likely associated with mobile device SLAM reconstruction and reduced GNSS reliability beneath dense vegetation cover [5,7,28]. Device orientation additionally appeared to influence survey consistency, with horizontally acquired surveys generally presenting lower rotational discrepancies relative to vertically acquired scans.

The observed limitations reinforce that mobile device LiDAR should not be interpreted as a replacement for survey-grade laser-scanning systems or high-precision geodetic surveys. Instead, the results suggest that the evaluated workflow may represent a practical and operationally accessible alternative for localized trail-condition assessment and exploratory geomorphological characterization, particularly in contexts where rapid data acquisition, low equipment costs, and ease of use are prioritized over absolute positional precision.

### 4.2. Diagnosis of Trail Degradation Features

Detailed validation performed using one representative aligned model demonstrated that the LiDAR-derived profiles reproduced the overall geometry of the analyzed trail tread, including incised sections, exposed rock surfaces, and localized surface irregularities. The extracted cross-sectional profiles additionally captured the asymmetrical morphology of the trail tread, characterized by multiple localized incision zones rather than a single centrally incised section. These results indicate that the evaluated smartphone LiDAR workflow can reproduce morphometric characteristics relevant to trail-condition diagnosis and localized erosion assessment.

Root characterization produced similar results. Width measurements derived from the LiDAR profiles showed comparatively low discrepancies relative to field observations, while protrusion measurements exhibited greater variability, particularly in transects characterized by abrupt microtopographic transitions and localized incision adjacent to the root system. Nevertheless, the extracted profiles consistently reproduced the general morphology and spatial variability of the exposed root feature.

From a trail-management perspective, these characteristics are particularly relevant because exposed roots, localized incision, uneven tread morphology, and other fine-scale surface irregularities directly influence localized slope variability, surface stability, visitor accessibility, and potential tripping hazards along the trail tread [13,14,17]. The ability to reproduce these features through rapid smartphone-based acquisition suggests that the evaluated smartphone LiDAR workflow may provide a useful tool for identifying degraded tread sections requiring maintenance or more detailed inspection [20,21].

The results additionally highlight an important methodological distinction between conventional field transects [12,13,14] and three-dimensional surface reconstruction approaches. Traditional cross-sectional surveys provide discontinuous measurements restricted to predefined transects and sampling intervals, whereas LiDAR-derived models preserve continuous three-dimensional surface geometry across the surveyed trail section. This continuity facilitates the identification of subtle microtopographic transitions, localized terrain irregularities, and preferential flow organization patterns that may remain partially unresolved in discrete manual measurements. Although the present study focused primarily on localized profile extraction, the generated three-dimensional models potentially support more extensive morphometric analyses, including longitudinal incision assessment and continuous terrain characterization along trail corridors.

The exploratory drainage and RSD analyses additionally demonstrated that the LiDAR-derived surface models preserve sufficient microtopographic detail to derive potential runoff pathways and topographic confinement zones along the trail tread. The derived potential runoff network exhibited strong spatial correspondence with the principal incised sectors of the trail, particularly along the dual-channel morphology identified on either side of the more elevated central rocky surface. Areas characterized by higher RSD values generally corresponded to sectors presenting more concentrated and continuous potential runoff organization, while more elevated and morphologically stable surfaces exhibited fewer or more fragmented flow paths.

The spatial correspondence observed between the potential runoff network, RSD patterns, and incised tread sectors further suggests that the reconstructed terrain captures topographic characteristics commonly associated with runoff concentration and trail degradation within the surveyed segment. These results suggest that the generated three-dimensional models may support not only morphometric characterization, but also exploratory assessments of potential runoff organization and erosion susceptibility along recreational trails.

### 4.3. Supporting Sustainable and Proactive Trail Management

The operational simplicity of the evaluated mobile device LiDAR suggests important potential applications for sustainable trail management. Data acquisition was rapid, required minimal equipment, and could be performed directly along the trail surface without specialized surveying procedures. These characteristics may facilitate more frequent and spatially distributed monitoring of trail conditions, particularly across recreational trail networks where conventional field surveys are difficult to maintain systematically due to time, staffing, and financial constraints [17,22].

The capability to generate three-dimensional representations of localized degradation features may additionally support maintenance prioritization and preventative management strategies. Trail incision, exposed roots, surface irregularities, and localized erosion features can directly affect visitor accessibility, safety, and trail sustainability [11,13,17]. The identification and monitoring of these features may therefore assist management authorities in identifying critical sections requiring intervention before more severe degradation processes develop.

The exploratory drainage and RSD analyses indicate that continuous surface models derived from mobile device LiDAR may help identify sectors associated with potential for runoff concentration and localized erosion development. This information may contribute to preventative maintenance planning by supporting the identification of poorly drained trail sections and areas where concentrated surface runoff may accelerate tread degradation.

The accessibility and increasing availability of LiDAR-enabled smartphones additionally raise the possibility of future decentralized and participatory trail-monitoring frameworks. Visitors, volunteers, or non-specialized personnel could potentially contribute localized three-dimensional surveys of degraded trail sections through dedicated digital platforms. Although such workflows were not directly evaluated in the present study, the results suggest that the evaluated mobile device LiDAR can produce morphologically meaningful representations of trail conditions with relatively limited technical requirements. This may create opportunities for expanding the spatial and temporal frequency of trail monitoring while reducing operational barriers associated with conventional surveying methods.

### 4.4. Limitations, Uncertainties, and Future Research

Several limitations should nevertheless be acknowledged. The study focused on a relatively short trail segment and a limited number of surveys performed under similar environmental conditions. The analysis emphasized localized morphometric characterization and representative profile validation rather than continuous longitudinal assessment of trail morphology along extended trail corridors. Dense canopy conditions prevented the acquisition of reliable differential GNSS control points, requiring manual alignment and preprocessing prior to inter-survey comparison. Although this limited the direct evaluation of absolute geospatial accuracy, repeated registrations demonstrated that the adopted alignment workflow produced consistent cloud-to-cloud comparison metrics, supporting the robustness of the preprocessing procedure for relative geometric analyses. Vegetation interference, localized reconstruction artefacts, and surface smoothing effects may additionally influence the representation of abrupt microtopographic transitions and highly irregular tread sections. Nevertheless, the resulting models consistently reproduced the principal morphological characteristics of the analyzed trail surface, including incision patterns, exposed roots, and localized surface irregularities. These limitations are consistent with previous studies highlighting the influence of acquisition conditions and vegetation cover on mobile device LiDAR reconstruction quality [7,8,18]. Similar methodological constraints and reconstruction uncertainties associated with mobile mapping systems have also been identified in broader reviews of mobile LiDAR applications [29].

The drainage and RSD analyses should additionally be interpreted as exploratory terrain representations derived from surface morphology rather than physically based hydrological simulations. Although the derived potential runoff patterns corresponded closely with visible tread incision and topographic depressions, additional validation under varying hydrological conditions would be necessary to evaluate the reliability of these approaches for predictive runoff or erosion modelling.

Future research should therefore explore the integration of mobile device LiDAR with continuous longitudinal profiling approaches capable of assessing incision variability along extended trail sections. Repeated temporal surveys may additionally help evaluate the applicability of the method for monitoring trail degradation dynamics and maintenance effectiveness through time. Additional comparisons against terrestrial laser scanning, survey-grade GNSS measurements, and independently georeferenced reference datasets under varying canopy and terrain conditions would also help clarify the operational limits, repeatability, and positioning consistency of mobile device LiDAR acquisition in recreational trail environments. Future studies could also explore the integration of continuous surface reconstruction with more advanced terrain and hydrological analyses, including temporal runoff monitoring, sediment redistribution assessment, and the identification of preferential erosion pathways along trail networks.

Overall, the results indicate that the evaluated smartphone LiDAR can provide operationally useful representations of hiking trail morphology and degradation features under realistic field conditions. While limitations related to positioning consistency, vegetation interference, and localized reconstruction artefacts remain present, the evaluated workflow demonstrates considerable potential as a rapid, accessible, and relatively low-cost approach for localized trail-condition assessment and provides a foundation for future research into smartphone-based trail monitoring.

## 5. Conclusions

This study evaluated the potential of a smartphone-based LiDAR workflow (iPhone 13 Pro and the Scaniverse application) for hiking trail assessment under densely vegetated field conditions. The results demonstrated that the evaluated workflow was capable of generating morphologically coherent three-dimensional representations of trail surfaces, including incised tread sections, exposed roots, rocky surfaces, and localized irregularities relevant to trail-condition assessment.

Despite variations in acquisition direction, phone orientation, and survey extent, repeated registrations demonstrated that the alignment workflow produced consistent cloud-to-cloud comparison metrics across the four surveys. Validation against field measurements additionally indicated that the LiDAR-derived profiles reproduced the general morphology of the analyzed trail surface and exposed root features with relatively low discrepancies.

The generated surface models additionally supported exploratory analyses of drainage organization and RSD patterns, supporting the identification of potential runoff pathways and topographic confinement zones associated with incised sectors of the trail tread. These results indicate that continuous three-dimensional surface reconstruction may support not only morphometric characterization, but also the exploratory interpretation of terrain conditions associated with erosion susceptibility and surface runoff concentration.

The study additionally demonstrated important operational advantages associated with mobile device LiDAR acquisition, including low equipment requirements, rapid field deployment, and the ability to conduct surveys directly beneath dense canopy cover where conventional differential GNSS positioning proved difficult to obtain. Although the technology does not replace survey-grade laser-scanning systems or high-precision geodetic surveys, the results suggest that it represents a practical and accessible alternative for fine-scale trail monitoring and localized degradation assessment.

Within the scope of this case study, the evaluated smartphone LiDAR workflow demonstrates that continuous three-dimensional characterization of hiking trail morphology is feasible using consumer-grade hardware. The results provide a foundation for further evaluation of smartphone LiDAR as a practical tool for localized trail-condition assessment and monitoring across a broader range of environmental conditions, devices, and trail settings.

## Figures and Tables

**Table 1:** Parameters for the four surveys done in the study area.

Parameter	Survey 1	Survey 2	Survey 3	Survey 4
Operator	User 1	User 2	User 1	User 2
Acquisition Time	00:01:20	00:03:30	00:01:47	00:02:08
Phone Orientation	Horizontal	Vertical	Horizontal	Vertical
Survey Direction	Descending	Descending	Ascending	Ascending
Approximate sensor distance to surface	<1 m	<1 m	>1 m	>1 m
Number of points	457,223	440,257	491,831	344,672

**Table 2:** Repeatability of cloud-to-cloud comparison results across three independent registrations. Values are the mean and standard deviation of the three repetitions. The relative range was calculated as the difference between the maximum and minimum values divided by the three-test mean.

Survey Pair	Mean Dist. (cm), Mean ± SD	Relative Range (%)	RMSE (cm), Mean ± SD	Relative Range (%)	95th Percentile (cm), Mean ± SD	Relative Range (%)	Points < 2 cm, Mean ± SD
S1-S2	1.10 ± 0.20	32.83	1.07 ± 0.22	36.37	2.76 ± 0.22	14.43	88.51 ± 4.49%
S1-S3	1.00 ± 0.17	33.73	1.23 ± 0.24	38.32	3.66 ± 0.80	42.91	89.38 ± 5.24%
S1-S4	1.90 ± 0.41	38.89	2.39 ± 0.56	44.80	7.59 ± 2.15	56.25	64.50 ± 10.40%
S2-S3	1.32 ± 0.09	12.70	1.53 ± 0.18	22.98	4.71 ± 0.51	21.64	79.69 ± 2.75%
S2-S4	1.84 ± 0.31	29.80	2.26 ± 0.44	35.30	8.10 ± 1.76	38.22	63.85 ± 7.75%
S3-S4	1.84 ± 0.36	36.42	2.33 ± 0.48	35.76	7.81 ± 1.80	42.53	65.79 ± 10.22%

**Table 3:** Statistical comparison between LiDAR-derived and field reference cross-sectional profiles on the measured transects.

Metric	Full Transect	Only Walkable Trail Path (≤1.6 m)	Outside Trail Path (>1.6 m)
MAE (cm)	4.11	3.83	5.08
RMSE (cm)	4.78	4.58	5.39
Mean Bias (cm)	4.11	3.83	4.91
Pearson Correlation (r)	0.86	0.81	0.95
Maximum absolute error (cm)	9.69	9.69	7.31

**Table 4:** Comparison between field reference and LiDAR-derived root width and protrusion measurements across the eight analyzed transects.

Metric	Width	Protrusion
MAE (cm)	0.53	2.9
RMSE (cm)	0.56	4.95
Mean Relative Error (cm)	0.13	0.44
Maximum absolute error (cm)	0.9	9.1
Minimum absolute error (cm)	0.2	0.3

## Data Availability

Data used for this study is available for downloading at the link: https://github.com/RuiMoraisFernandes/mobile-LiDAR-trails/tree/main (accessed on 4 July 2026).

## Supplementary Materials

The following supporting information can be downloaded at: https://www.mdpi.com/article/10.3390/s26165143/s1, Tables S1–S3: Full cloud-to-cloud comparison statistics between the four mobile LiDAR surveys, showing inter-survey geometric consistency and point-distance agreement thresholds.

## Abbreviations

The following abbreviations are used in this manuscript:

DSM	Digital Surface Model
LiDAR	Light Detection and Range
RSD	Relative Surface Depression
