# Supplementary material for: Potential of Mobile LiDAR Sensors in Hiking Trail Management
Source: Sensors (Basel). 2026 Aug 14;26(16):5143. doi: 10.3390/s26165143 (PMC13517338; doi:10.3390/s26165143)
Supplement: Supplementary file 1 [file sensors-26-05143-s001.zip › Table S1.pdf]

sensors

MDPI

Table S1. Full cloud-to-cloud comparison statistics between the four mobile LiDAR surveys, showing inter-survey geometric consistency and point-distance agreement thresholds (Test 1).

Stat	1v2	1v3	1v4	2v3	2v4	3v4	Mean of stat
Max (cm)	0.207942	0.271568	0.39146	0.222226	0.372449	0.319594	0.29753983
Min (cm)	0.000036	0.000102	0.000149	0.000079	0.000107	0.00014	0.00010217
Average (cm)	0.00968	0.01163	0.02165	0.013642	0.021915	0.019897	0.0164021
Median (cm)	0.006712	0.008682	0.016521	0.010687	0.017675	0.014798	0.0125125
Std. Dev. (cm)	0.009997	0.009138	0.018199	0.009825	0.016964	0.016933	0.01350938
Percentile	0.026129	0.04542	0.095987	0.047726	0.09065	0.086021	0.0653223
<1 cm	71.356	56.70332	30.43738	46.98067	28.04862	34.53524	44.6768717
<2 cm	91.32364	83.76269	59.11156	78.41113	56.07028	63.33389	72.0021962
<5 cm	99.11294	100	91.40443	100	92.41883	92.62571	95.9269841
RMSE (cm)	0.009381	0.014791	0.028283	0.014761	0.027713	0.026127	0.02017585
Count Raw (n)	304713	310979	202981	310979	202981	202981	-
Count Filter (n)	289476	295018	192832	295430	192833	192832	-
Points Retained (%)	94.99956	94.8675	95.00002	94.99998	95.00052	95.00002	-
