# Supplementary material for: Potential of Mobile LiDAR Sensors in Hiking Trail Management
Source: Sensors (Basel). 2026 Aug 14;26(16):5143. doi: 10.3390/s26165143 (PMC13517338; doi:10.3390/s26165143)
Supplement: Supplementary file 1 [file sensors-26-05143-s001.zip › Table S2.pdf]

sensors

MDPI

Table S2. Full cloud-to-cloud comparison statistics between the four mobile LiDAR surveys, showing inter-survey geometric consistency and point-distance agreement thresholds (Test 2).

Stat	1v2	1v3	1v4	2v3	2v4	3v4	Mean of stat
Max (cm)	0.332666	0.273636	0.277754	0.287318	0.369456	0.350361	0.315199
Min (cm)	0.000112	0.000093	0.000245	0.000074	0.00006	0.00022	0.000134
Average (cm)	0.009978	0.008258	0.020959	0.012078	0.01682	0.021081	0.014862
Median (cm)	0.006765	0.006361	0.01709	0.009284	0.01526	0.017211	0.011995
Std. Dev. (cm)	0.012227	0.005744	0.015286	0.008661	0.01033	0.01541	0.011276
Percentile	0.026579	0.029713	0.078395	0.041636	0.091669	0.090751	0.05979
<1 cm	70.36064	71.86986	28.53781	53.57962	32.78859	28.40814	47.59078
<2 cm	90.86743	94.14474	57.89543	82.85642	63.92936	57.02194	74.45255
<5 cm	99.08081	100	93.28319	100	100	93.6368	97.6668
RMSE (cm)	0.00957	0.010059	0.025941	0.013836	0.019738	0.026113	0.017543
Count Raw (n)	300046	300046	300046	306501	200122	306501	-
Count Filter (n)	285043	285042	285043	291175	190112	291175	-
Points Retained (%)	94.99977	94.99943	94.99977	94.99969	94.99805	94.99969	-

 https://doi.org/10.3390/s26165143
