# Supplementary material for: Potential of Mobile LiDAR Sensors in Hiking Trail Management
Source: Sensors (Basel). 2026 Aug 14;26(16):5143. doi: 10.3390/s26165143 (PMC13517338; doi:10.3390/s26165143)
Supplement: Supplementary file 1 [file sensors-26-05143-s001.zip › Table S3.pdf]

sensors

MDPI

Table S3. Full cloud-to-cloud comparison statistics between the four mobile LiDAR surveys, showing inter-survey geometric consistency and point-distance agreement thresholds (Test 3).

Stat	1v2	1v3	1v4	2v3	2v4	3v4	Mean of stat
Max (cm)	0.165728	0.285548	0.273744	0.665115	0.642797	0.260374	0.382218
Min (cm)	0.000181	0.000121	0.00009	0.000104	0.000187	0.000157	0.00014
Average (cm)	0.013285	0.010106	0.014276	0.013749	0.016434	0.014363	0.013702
Median (cm)	0.010845	0.008382	0.011149	0.010752	0.013482	0.011188	0.010966
Std. Dev. (cm)	0.010645	0.006819	0.010231	0.010592	0.011966	0.01048	0.010122
Percentile	0.030113	0.034693	0.053298	0.051821	0.06071	0.057533	0.048028
<1 cm	45.47361	60.37222	45.01726	47.10271	36.8259	44.59012	46.56364
<2 cm	83.33711	90.24707	76.49404	77.81527	71.56384	77.02251	79.41331
<5 cm	98.75358	100	100	99.60286	97.53722	99.84396	99.2896
RMSE (cm)	0.01329	0.012191	0.017564	0.017356	0.020328	0.01778	0.016418
Count Raw (n)	309099	309099	309099	202766	202766	314861	-
Count Filter (n)	293644	293644	293644	192627	192627	299117	-
Points Retained (%)	94.99998	94.99998	94.99998	94.99965	94.99965	94.9997	-
